# Evidence of visual crossmodal reorganization positively relates to speech outcomes in cochlear implant users

**DOI:** 10.1038/s41598-022-22117-z

**Published:** 2022-10-22

**Authors:** Brandon T. Paul, Münir Demir Bajin, Mila Uzelac, Joseph Chen, Trung Le, Vincent Lin, Andrew Dimitrijevic

**Affiliations:** 1 Department of Psychology, Toronto Metropolitan University, Toronto, ON M5B 2K3 Canada; 2 Otolaryngology—Head and Neck Surgery, Sunnybrook Health Sciences Centre, Toronto, ON M4N 3M5 Canada; 3 Evaluative Clinical Sciences Platform, Sunnybrook Research Institute, Toronto, ON M4N 3M5 Canada; 4 Faculty of Medicine, Otolaryngology—Head and Neck Surgery, University of Toronto, Toronto, ON M5S 1A1 Canada

**Keywords:** Cognitive neuroscience, Human behaviour

## Abstract

Deaf individuals who use a cochlear implant (CI) have remarkably different outcomes for auditory speech communication ability. One factor assumed to affect CI outcomes is visual crossmodal plasticity in auditory cortex, where deprived auditory regions begin to support non-auditory functions such as vision. Previous research has viewed crossmodal plasticity as harmful for speech outcomes for CI users if it interferes with sound processing, while others have demonstrated that plasticity related to visual language may be beneficial for speech recovery. To clarify, we used electroencephalography (EEG) to measure brain responses to a partial face speaking a silent single-syllable word (visual language) in 15 CI users and 13 age-matched typical-hearing controls. We used source analysis on EEG activity to measure crossmodal visual responses in auditory cortex and then compared them to CI users’ speech-in-noise listening ability. CI users’ brain response to the onset of the video stimulus (face) was larger than controls in left auditory cortex, consistent with crossmodal activation after deafness. CI users also produced a mixture of alpha (8–12 Hz) synchronization and desynchronization in auditory cortex while watching lip movement while controls instead showed desynchronization. CI users with higher speech scores had stronger crossmodal responses in auditory cortex to the onset of the video, but those with lower speech scores had increases in alpha power during lip movement in auditory areas. Therefore, evidence of crossmodal reorganization in CI users does not necessarily predict poor speech outcomes, and differences in crossmodal activation during lip reading may instead relate to strategies or differences that CI users use in audiovisual speech communication.

## Introduction

Auditory deprivation, such as in deafness or hearing loss, can drive a *crossmodal* form of neuroplasticity in auditory cortex where these regions reorganize to support non-auditory sensory functions such as vision^1^. Crossmodal reorganization may arise through unmasking of latent neuronal inputs, axonal sprouting, increased dendritic branching, and increased synapse numbers in deprived auditory regions, or changes in the balance of sensory input from subcortical areas or through cortico-cortical connections^2–4^. Crossmodal plastic changes may account for instances of “supra-normal” perception found in deaf individuals, such as improved visual motion perception and peripheral attention compared to hearing controls^5–7^. The extent to which deprivation modifies the structure or function of human auditory cortex is not clear, but recent theories suggest a “functional remapping” account where deafferented cortical regions preserve their functional role when responding to an intact sense (e.g., cortical regions that support motion processing in audition now support visual motion processing in deafness^4^), or entirely change their function (e.g., auditory cortex contributing to visual working memory in deafness^8^) or a combination of preserved function and functional change (see^9,10^ for reviews).

Crossmodal reorganization following deafness is viewed as an adaptive phenomenon because the upregulation of visual processing compensates for a loss of hearing^11^. However, it is necessary to consider how this plasticity affects sound processing if the auditory system is restored using a cochlear implant (CI). CIs are surgically implanted devices used in cases of deafness or profound hearing loss where hearing aids do not provide sufficient benefit. CIs transduce acoustic signals into electrical pulse trains and deliver them directly to the auditory nerve across several frequency channels, and while speech signals are spectrally degraded, they usually provide enough fidelity for auditory speech communication. Successful speech communication with a CI depends on multiple factors such as duration and degree of deafness, age at implantation and language development stage, effect of etiology, and other variables^12,13^. Crossmodal plasticity is another factor that can predict speech outcomes, and CI users show evidence of enhanced crossmodal activation to visual stimuli both before^14^ and after implantation^15,16^. Deprived auditory cortical neurons that begin to preferentially respond to vision or touch might not be available to process sound information if the auditory system is reafferented, establishing a *competition* between auditory- and visual-responding neurons for sensory processing^17^.

Studies that find poorer speech outcomes in CI users with more extensive crossmodal reorganization in auditory areas support a competition viewpoint. For instance, Lee and colleagues^14^ used PET to measure resting-state metabolic activity in auditory cortical regions (Brodmann areas 41, 42, and 22) in prelingually deaf children and young adults before cochlear implantation. Participants with higher auditory cortical metabolism had lower speech ability measured 8 to 44 months after CI activation, and metabolic activity was a stronger predictor of speech ability than duration of deafness and CI use. The authors argued that visual or somatosensory crossmodal plasticity restored metabolism in the deprived auditory cortex during deafness, leaving auditory cortex less responsive to incoming signals from the CI (see also^18,19^). Strelnikov et al.^20^ similarly found that stronger resting-state metabolic activity in temporal cortex, as well as hemodynamic responses to visual and audiovisual words, were negatively correlated with auditory performance scores. Larger evoked responses could reflect visual cross-modal reorganization in auditory areas during deafness, which leaves fewer resources available for processing auditory signals once the ear is reafferented by the cochlear implant.

Past research in CI users relied on visual evoked potentials (VEPs) to track crossmodal effects due to the compatibility of electroencephalography (EEG) with the implanted device. In line with a competition viewpoint, these correlational studies found lower speech performance in individuals with larger or earlier visual evoked potentials sourced to (mostly right) auditory cortex or in scalp locations that suggest involvement of temporal cortex during visual processing. The effects appear in different populations and with different stimuli, for instance, in prelingually deafened adults and children using visual apparent motion^21^ or visual gradients^22^, and in postlingually deafened adults using apparent motion stimuli^15^ or inverting checkerboards^23,24^.

Not all studies agree that visual crossmodal plasticity in CI users is detrimental for speech outcomes. Anderson and co-authors^25^ used visual and audiovisual speech stimuli to track changes in crossmodal plasticity before and after implantation in a group of mostly postlingually deafened adults. They found that visual activation of superior temporal cortex was associated with improved speech outcomes and stronger auditory responses in CI users, challenging previous findings that link poor speech outcomes with visual crossmodal plasticity. The results were consistent with experiments in animal models showing that reorganization of higher-order auditory regions does not reduce auditory responsiveness and is unlikely to negatively interfere with speech recovery^26^. These findings implicate a *cooperation* viewpoint where plasticity related to visual language or visual communication assists speech recovery, either by helping individuals effectively use visual cues in auditory communication or by promoting audiovisual integration during speech listening^27,28^. In this way, relative differences in visual plasticity between CI users with higher or lower speech outcomes may reflect the degree to which CI users rely on or integrate visual language information with auditory input, rather than plasticity interfering with auditory speech processing.

We previously tested the idea that large crossmodal activations to language-relevant visual stimuli would predict positive speech outcomes in CI users by examining EEG responses to visually presented letters and numbers in a modified Sternberg working memory task. Although we found evidence for modified brain connectivity during working memory and stronger visual responses in auditory cortical regions in CI users compared to age-matched controls, the responses did not correlate to speech outcomes^29^. We also found that the power of 8–12 Hz (alpha) brain oscillations arising 300–400 ms after the onset of the visual characters was lower for CI users than age-matched typical-hearing controls. One interpretation of this finding is that task-related rhythmic alpha activity in M/EEG recordings arises from inhibitory cortical interneurons that perform sensory gating^30^. If larger decreases in alpha power reflect less inhibition, the result suggests a facilitation of visual character processing in CI users’ sensory networks. Oscillatory responses may afford a novel characterization of crossmodal effects on visual stimulus processing in deafness and hearing loss.

The goal of the current study was to compare speech performance outcomes to evidence of crossmodal reorganization in a group of CI users with a visual speech (lipreading) stimulus and lipreading task, because auditory and visual integration is more likely for these stimuli compared to orthographic characters our group used previously^29^. We recorded multichannel EEG and used source analysis methods to analyze two types of visual responses in auditory cortex while participants performed the lipreading task: the response to the onset of the video clip featuring a face, and ongoing neural oscillations during the segment of the video clip where the lips were moving. We opted to use face responses and a lipreading task because CI users are known to show elevated responses in auditory regions to face stimuli^16,31^. This past work found no differences in face recognition between CI users and controls, but the strength of auditory cortex activation to faces positively predicted face recognition performance^16^ and stronger audiovisual integration^31^. The results suggest that elevated responses to face stimuli in auditory regions are related to behavioural outcomes in CI users.

We hypothesized that (1) CI users will have higher lipreading accuracy compared to typical hearing controls, consistent with past studies^16,28,31–33^, (2) brain responses to the onset of the lipreading video and during lip movement would be significantly different between CI users and controls in auditory cortical regions as evidence of crossmodal reorganization, and (3) crossmodal brain responses in auditory cortex would positively relate to speech-in-noise (SIN) listening ability, consistent with the *cooperation* view of sensory restoration and crossmodal plasticity.

## Material and methods

### Participants

Fifteen adult CI users with at least 1-year of CI experience were recruited through the Sunnybrook Health Sciences Cochlear Implant Program whose age ranged from 18 to 79 years (mean ± SD; 57 ± 20 years). All but four CI users were postlingually deafened. Demographics shown in Table 1. Additionally, a control group of 13 adults with age-appropriate normal hearing (NH), whose age was matched to CI users and ranged from 18 to 78 years (mean ± SD; 55 ± 21 years) participated in the study. No participants reported a history of sign language use.

**Table 1:** Clinical characteristics of CI participants.

ID	Age	Sex	Deafness onset age	Deafness duration (yrs)	Deafness etiology	CI Side	Other device	CI use (yrs)		AzBio + 5 dB SNR (%)
								Left	Right	
CI01	18	M	Birth	18	Unknown	Bilateral	–	3	15	83
CI02	25	F	Birth	25	Hereditary	Right	None	–	20	40
CI03	28	F	3	26	Unknown	Right	None	–	16	0
CI04	54	M	9	45	Suspected Hereditary	Right	None	–	2	40
CI05	54	M	22	32	Suspected Hereditary	Right	Right HA	–	2	67
CI06	60	F	Birth	60	Suspected Hereditary	Bilateral	–	10	17	15
CI07	62	F	15	47	Unknown	Right	Left HA	–	6	22
CI08	62	M	Birth	62	Hereditary	Left	Right HA	13	–	20
CI09	63	M	40	23	Meniere's disease	Left	Right HA	4	–	76
CI10	63	M	16	47	Bilateral Otosclerosis	Right	Left HA	–	2	82
CI11	71	M	56	15	Unknown	Left	Right HA	6	–	47
CI12	72	M	61	12	Unknown	Left	Right HA	1	–	52
CI13	74	F	48	26	Suspected Hereditary	Right	Left HA	–	4	70
CI14	74	M	55	20	Unknown	Left	Right HA	11	–	9
CI15	78	M	70	9	Hereditary/Noise exposure	Left	None	1	–	25

*CI* cochlear implant, *HA* hearing aid, *AzBio* Arizona Biomedical Institute Sentence List, *dB* decibels, *SNR* speech-to-noise ratio, *yrs* years.

All participants were informed verbally and in writing of all experimental procedures and provided written informed consent. The methods used in this study were approved and performed in accordance with the guidelines and regulations outlined by the Research Ethics Board (REB) at Sunnybrook Health Sciences Centre (#474–2016) and accorded with the Declaration of Helsinki. Participants were compensated with money for their participation and were provided full reimbursement for parking at the hospital campus.

### Audiologic testing

Speech-in-noise (SIN) testing using the Arizona Biomedical Institute (AzBio) sentence list^34^ was performed in CI users in a sound booth with a single speaker at 0° azimuth 1.5 m away from the subject. The AzBio sentence list was presented at 65 dB sound pressure level (SPL) while speech-shaped noise was presented at 60 dB SPL yielding a speech-to-noise ratio (SNR) of + 5 dB. CI users listened to fifty sentences, and their score was tallied as the percent of correctly identified words. SIN scores were not tallied for control subjects who typically perform the AzBio task at ceiling.

### Stimuli

Prior to performing any tasks, participants were provided a list of 46 monosyllabic words for two minutes. Not all words were included as experimental stimuli. While seated 1 m from the visual display monitor, participants were first presented with a 2.0 s crosshair fixation task followed by a 2.5 s silent video clip of a male articulating a monosyllabic word. The video was cropped below the eyes showing only the nose, lips, and chin. The same set of stimuli were used in a previous study examining audiovisual integration and event-related potentials^35^. Stimulus presentation was followed by a fillable textbox where participants were asked to type the perceived word and indicate the confidence of their response on scale of 1 (“not confident at all”) to 10 (“absolutely confident”). Twenty such trials comprised one block, and a total of 10 blocks were presented for a total of 200 trials. Participants were encouraged to take a break after each block.

### EEG recording

The 64-channel EEG was sampled at 2000 Hz using an actiCHamp Brain Products system (Brian Products GmbH, Inc., Munich, Germany) coupled to a Neuroscan amplifier. The electrode cap employed equidistant electrode placement with the reference channel at the vertex (sensor Cz) and the ground electrode 50% of the distance to the nasion. The 3D locations of each electrode were recorded using a Polhemus FastTrack digitizer (Colchester, USA).

### Data processing

Brain Vision Analyzer v2.0 (Brain Products GmbH Inc., Munich, Germany) was used to analyze electrophysiologic data using a 0.01 Hz high-pass filter to remove baseline drift. Subsequent down-sampling of data to 500 Hz was performed. Any extreme stereotypical participant movement artifacts (≥ 500 mV) were removed. Ocular, cardiac, and transient electrode artifacts were removed with the Brain Vision Analyzer Independent Component Analysis (ICA), which employs an identical (ICA) algorithm to EEGLAB^36^. Continuous data were then exported to the Brainstorm toolbox^37^ in MATLAB for further analysis.

### Visual evoked potentials (VEPs)

Continuous EEG data were averaged referenced and segmented from − 500 to 3000 ms in order to capture the onset of the face from the movie stimuli at 0 ms and the end of the movie clip at 2500 ms. The global field power (GFP) was calculated by taking the standard deviation across channels for each time point. The grand-average GFP was used to identify the P1/N1 peak, and separate 20 ms windows centered on P1/N1 were used to extract averaged values for each of the CI and NH participants.

### Source analysis of VEPs

Sources of VEPs were computed using standardized low-resolution electromagnetic tomography (sLORETA) modelling^38,39^ using the default settings in Brainstorm. A boundary element model (BEM) head model was created with the OpenMEEG plugin in Brainstorm. Each sLORETA map was used to extract the absolute values of the source time series (aka ‘scouts’) in predefined regions of interest (ROIs) of bilateral auditory cortices based on the Desikan–Killiany atlas^40^ following regions suggested by Stropahl et al.^31^ and recently reported in our VEPs to a visual working memory in CI users^29^. The auditory ROI was selected to approximate Brodmann areas 41 and 42. The visual cortices were based on “V1” Brodmann areas in Brainstorm, corresponding to Brodmann area 17. A “Face Area” ROI was determined based on maximum sLORETA N1 activation and was in the location of previously described occipital face areas near Brodmann area 37. Group comparisons were performed on the ROIs’ time-waveform activation on a 20-ms time window centred on the N1 peak amplitude.

### Time–frequency analysis

Continuous EEG data were averaged referenced, segmented from − 1000 to 5000 ms and subjected to time–frequency analysis in Brainstorm. Default Brainstorm time–frequency Morlet wavelets were applied (1 Hz central frequency, and full width half maximum at 3 s). Frequency power was computed for 1 to 40 Hz in 1 Hz steps. After computing the time–frequency maps, data were normalized using event-related desynchronization/synchronization (ERD/S)^41^ calculation in Brainstorm as follows: ERD/S = (power-mean baseline power)/ mean baseline power × 100 where the baseline was − 1000 to − 4 ms.

### Sources of ERS/ERD

Source reconstruction for time–frequency data was performed in Brainstorm using default settings for the LCMV beamformer. Time–frequency time bins were chosen based on grand-average time–frequency plots for the auditory and visual ROIs. The same auditory and visual ROIs used for imaging the N1 onset were used for the LCMV beamformer.

### Statistical analysis

Statistical analyses were performed in MATLAB 2018a using the Statistics and Machine Learning Toolbox and in the R statistics package^42^. In R, behavioural performance and sensor-level potentials were analyzed using unpaired t-tests. In cases where data were not normally distributed, Wilcoxon rank sum tests were used. Source-space responses were first compared between CI and NH groups within each ROI using cluster-based permutation testing in Brainstorm (5000 permutations). We then followed up by comparing onset responses across ROIs using mixed analysis of variance (ANOVA) within the *afex* package in R. Post-hoc comparisons were assessed using the *emmeans* package and were corrected for false discovery rate (FDR).

Pearson correlations were used compare behavioural performance to brain activity, and Spearman’s rho was used in instances where data were not normally distributed. Planned correlation analysis included comparison of CI users’ AzBio (SIN) scores to activity in auditory ROIs during the onset of the video stimulus, and during the lip movement period. Correlations between performance and demographic variables were exploratory across neural responses and were corrected for FDR.

The alpha criterion for all tests was set to 0.05, and all tests were two-tailed. Test statistics are also reported alongside eta squared (*η*^2^) values (generalized eta squared for ANOVA models) to indicate effect sizes, with conventions of 0.01 as a small effect, 0.06 a medium effect, and 0.14 and above as large effects.

## Results

### Task performance, response confidence, and CI

CI users’ average word identification accuracy (31.7%, SD = 16.0) did not differ from the NH group (24.2%, SD = 11.1; p = 0.24), and confidence ratings also did not differ (M(SD) for CI = 6.0(1.9); NH = 5.1(1.7); p = 0.19). Across all participants, individuals who were more confident in their answer had higher performance (r = 0.51, p = 0.005).

CI users’ SIN ability (AzBio-in-noise scores) was not related to their performance on the lipreading task or confidence ratings (p’s > 0.30). Duration of deafness and age at deafness onset did not correlate to task performance or confidence (p’s > 0.20) but CI users’ maximum duration of CI use (i.e., the duration of use for the first implanted CI in the case of bilateral users) was positively correlated to task performance (r = 0.55, p = 0.032).

### Sensor event-related potentials

**Figure 1:**
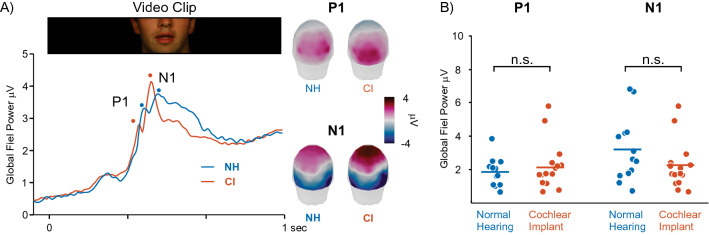
EEG sensor results. (**A**) Grand average EEG global field power during the lipreading video clip for NH (blue) and CI (red) groups. The video clip began at 0 s and ended at 2.5 s as bounded by the still image from an example clip above the time series. The topographic plots in the right side of panel A are from a posterior view and show voltage distributions at the P1 and N1 GFP peaks for both groups. (**B**) NH and CI group differences in GFP at P1 and N1 peaks. Each dot represents a participant. For all scatterplots, colored horizontal lines reflect group means.

The grand average VEP expressed as global field power (GFP, the spatial standard deviation; Fig. 1A) demonstrates the characteristic P1/N1 response to the onset of the movie, and the morphology and topography these responses were similar between CI and NH groups. P1 response voltages were expectedly positive at occipital sensors while N1 responses were negative toward parietal sensors. CI and NH groups did not differ in P1 (p = 0.08) or N1 (p = 0.90) GFP magnitude (Fig. 1B).

### Source event-related potentials

**Figure 2:**
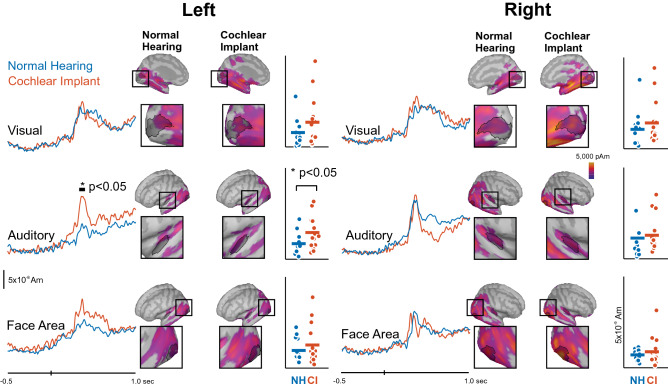
For left and right visual, auditory, and face ROIs illustrated as outlined regions on brain templates, sLORETA activations fit to the peak N1 GFP response following the video onset are shown as a time series comparing CI users (red) and NH controls (blue). ROI boundaries for each region on left and right sides are shown within brain plots shaded with higher activation toward yellow and lower activation toward purple. For each region, individual differences for each group are shown as scatterplots in the rightmost panels. For all scatterplots, colored horizontal lines reflect group means.

To test for evidence of crossmodal reorganization, we used sLORETA to identify sources contributing to the N1 onset response in ROIs that covered auditory, visual, and face regions for left and right hemispheres. Figure 2 shows the time course of activation, location of each region, and subject-level data for each ROI. Nonparametric cluster-based permutation tests were first used to compare the time series of activation between groups at each ROI. The only difference was identified for the left auditory ROI, suggesting stronger N1-like onset responses in CI users compared to controls (p < 0.05). We then used a 2 × 2 × 3 ANOVA to compare onset activation magnitudes between groups for within-subjects factors of hemisphere (left vs. right) and ROI (auditory, visual, face). No significant main effects or interactions were identified (p’s > 0.10). Although isolated t-tests on each region suggested a group difference in the left auditory ROI, this result does not hold when compared to other brain regions.

### Changes in cortical alpha power during lipreading

**Figure 3:**
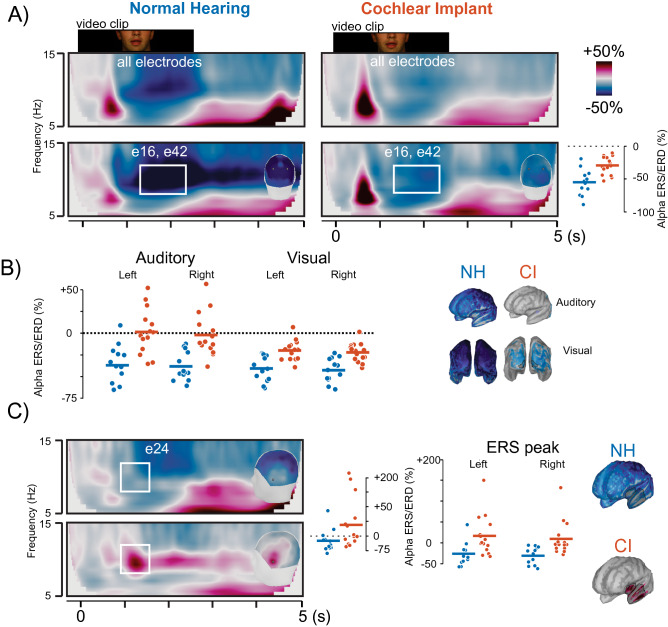
Neural oscillation analysis. (**A**) EEG event-related synchronization and desynchronization (ERS/D) across frequencies expressed as a percent change of power from baseline for all electrodes (top row) and occipital sensors e16 and e42 roughly approximating channels O1 and O2 as seen on 3D head shapes within time frequency plots (bottom row). The Scatterplot in  the rightmost panel compares ERS/D for CI (red) and NH (blue) groups in the time frequency range outlined in the bottom row (white box) for 8 to 12 Hz alpha power from ~ 1.3 to 2.3 s where the ERD was at maximum. (**B**) Activity in auditory and visual ROIs in left and right hemispheres for the source of the ERD is shown for individual subjects in CI (red) and NH (blue) groups. Whole-brain plots show distributions of alpha power ERS/ERD from left frontal (top) and posterior (bottom) views. (**C**) CI users produced an alpha ERS in grand average plots across sensor e24 (approximating FT7). Individual differences in ERS/D for both groups are plotted in as scatters in the middle panel for sensor e24, as well as bilateral regions of superior temporal cortex where sources of alpha ERS were found in CI users (rightmost panel). For all scatterplots, colored horizontal lines reflect group means.

Changes in the power of neural oscillations during the video clip are shown in Fig. 3A. Averaging across all channels, we observed increases in power relative to baseline from 6 to 12 Hz in all participants after the onset of the film from before 1 s, followed by a strong ERD of alpha (8–12 Hz) power across the scalp from 1 to 3 s (Fig. 3A, top row). The decrease in alpha power appeared comparatively smaller in CI users and was significantly different when averaging over occipital sensors where the ERD was strongest (t(26) = 4.11, p = 0.0003, η^2^ = 0.39; Fig. 3A, bottom row). A 2 × 2 × 2 ANOVA compared between-group differences for alpha ERD from 1.3 to 2.3 s in auditory and visual ROIs using within-subjects factors of hemisphere (left vs. right) and ROI (auditory vs. visual) in the model. We found main effects of group (F(1,26) = 26.28, p < 0.001, η^2^ = 0.40) and cortex (F(1,26) = 28.92, p < 0.001, η^2^ = 0.21) as well as an interaction between group and cortex (F(1,26) = 5.55, p = 0.026, η^2^ = 0.05). The three-way interaction between sensory cortex, group, and hemisphere was not significant (p = 0.78). FDR-corrected post-hoc tests, collapsing across hemisphere, found less ERD in the CI group in visual cortex (p < 0.001) and auditory cortex (p = 0.004). Together these results suggest weaker ERD and a mixture of ERS and ERD in the CI group during movie watching compared to NH individuals who had stronger and more consistent ERD in visual and auditory ROIs.

Inspection of individual differences in activation for auditory cortex sources suggested that some CI users produced ERS when watching the movie clip. In Fig. 3C, scalp topographies showed that this ERS was situated along left temporal channels and reaching a maximum in channel e24 (comparable to FT7) and peaked from 1 to 1.5 s. ERS was significantly higher at this location and time in CI users (Wilcoxon rank-sum test, W = 148, p = 0.021). Sources of this alpha ERS localized to left temporal cortex. Group comparison of this region in the left and right hemisphere were assessed in a 2 × 2 mixed ANOVA. The model returned a main effect of group (F(1,26) = 9.85, p = 0.004, η^2^ = 0.26) and no main effect of hemisphere or interaction with group (p’s > 0.15). The results suggest a tendency toward alpha ERS in some CI users during movie watching in temporal areas, which is opposed to alpha ERD that was more common for NH listeners.

### Brain-behaviour correlations

#### Relationship of brain activity to task performance

We compared lipreading performance and response confidence to onset responses and alpha oscillation power during lipreading in both sensor and source space. After correcting p-values for multiple comparisons, there were no significant correlations. Uncorrected, there was a negative correlation between N1 onset activation averaged across left and right auditory ROIs to behavioural response confidence, where larger responses were associated with lower confidence (r =  − 0.48).

#### Relationship of brain activity to speech-in-noise listening performance

**Figure 4:**
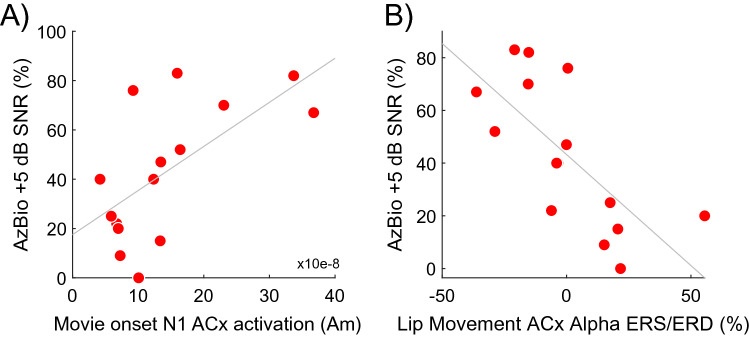
Correlations of crossmodal activity and speech scores. (**A**) Visual crossmodal activation of auditory cortex to the movie onset positively predicts speech-in-noise performance in CI users measured by AzBio sentences in speech-shaped background noise at + 5 dB SNR. (**B**) Alpha synchronization and desynchronization during speech-related lip movement expressed as a percent change in 8 to 12 Hz power from baseline negatively predicts speech-in-noise performance.

Because the lipreading stimulus has relevance for language and communication, we hypothesized a positive relationship (e.g., *cooperation* model) between activation of auditory cortex to the onset of the lipreading movie and speech-in-noise listening ability. This prediction was confirmed (Fig. 4A). Larger N1 onset responses localized to auditory ROIs and averaged across cortex predicted higher SIN listening scores measured using AzBio sentences in background noise at + 5 dB SNR in CI users (r = 0.63, p = 0.011). Although crossmodal responses in CI users were not significantly larger than NH controls when compared across brain regions, larger crossmodal activations were less characteristic of the NH group and the direction of results run counter to the competition view that larger auditory cortex responses to visual stimulus onset are related to lower speech scores in CI users.

We then compared CI users’ SIN performance to beamformed alpha activity in an average of left and right auditory ROIs (Fig. 3B), where there was a mixture of ERS and ERD during lipreading. Shown in Fig. 4B, CI users with more ERS during lipreading had comparatively lower SIN ability, while CI users with more ERD had higher SIN performance (r =  − 0.71, p = 0.003). This contrasts with the correlation found for the movie onset response in auditory ROIs, in that brain activity during lip movement that was more dissimilar to NH controls (i.e., more ERS) was associated with lower SIN performance. These diverging relationships would also suggest that CI users with larger onset responses had stronger ERD in auditory ROIs during lipreading, which was confirmed by a negative correlation between the two responses (r =  − 0.63, p = 0.012). NH controls showed the opposite relationship, with larger onset responses relating to *less* ERD in auditory ROIs (Spearman’s *rho* = 0.52, p = 0.07). The correlation between onset response magnitude and alpha ERS/ERD was significantly different between groups (Fisher’s r-to-z =  − 3.08, p = 0.002). Finally, we note that the relationship of SIN ability to onset responses and alpha ERS/D in auditory ROIs was not mediated by CI users’ age, duration of deafness, maximum duration of CI use, or age of deafness onset. This suggests that CI users with congenital deafness were not the individuals driving these correlations. We also did not find any significant relationship between SIN ability and visual onset responses or alpha ERS/ERD in visual sources (uncorrected p’s > 0.08).

In sum, CI users with lower SIN ability tended to have smaller movie-onset responses in auditory ROIs but produced higher alpha ERS in auditory ROIs while viewing the lip movement. CI users with higher SIN ability had larger onset responses and produced alpha ERD during lip movement in auditory ROIs. In other words, CI users that were dissimilar to NH listeners in onset responses (more crossmodal activation) but similar during lip movement had higher SIN scores. CI users with similar onset responses to NH controls (less crossmodal activation) but dissimilar activity during lip movement had lower SIN scores.

#### Relationship of brain activity to duration of deafness and age at deafness onset

Finally, we explored how the duration of deafness and the age at deafness onset in CI users related to brain activity at video onset and during lip movement. CI users with earlier ages of deafness onset had larger N1 responses at the sensor level (r =  − 0.63), but this did not survive FDR correction (p = 0.12). There were no significant correlations between duration of deafness and brain responses (uncorrected p’s > 0.052).

## Discussion

### Summary of findings

CI users’ speech listening outcomes are highly variable^13^ and visual crossmodal reorganization in auditory cortex may partially account for these individual differences^27^. Using EEG, we measured visual crossmodal activation of auditory areas while a group of primarily postlingually deafened CI users and age-matched NH controls performed a lipreading task with monosyllabic words. CI users did not differ from the NH group in lipreading performance or their response confidence ratings, and in CI users, lipreading performance was not related to age, duration of deafness, or age at deafness onset. However, performance was positively correlated to duration of CI use.

We found differences in CI users’ brain responses to the lipreading video clip. Upon video onset, CI users did not have overall larger responses at the sensor level or source level in visual cortex or face areas compared to controls, but CI users had a stronger activation of left auditory cortex when this region was tested in isolation. While observing lip movement, alpha oscillation power decreased relative to baseline in auditory and visual areas in NH controls, but CI users showed a mixture of increases (ERS) and decreases (ERD) relative to baseline in these regions. CI users with ERS tended to have sources in left temporal cortex that overlapped with speech processing areas, including superior temporal cortex. Notably, the differences in brain responses between CI users and NH controls for the lipreading stimulus related to CI users’ clinical speech-in-noise (SIN) listening tests. CI users with higher SIN performance had a larger onset response in auditory cortex but also produced more ERD in auditory areas during lipreading. In contrast, CI users with relatively lower SIN performance had weaker onset responses and produced more ERS during lipreading. The findings overall suggest that visual crossmodal activity in CI users has starkly different relationships to SIN ability depending on the type of visual response.

### No differences in lipreading performance between CI users and age-matched controls

Previous research suggests that CI users’ reliance on visual cues for language understanding or audiovisual integration accounts for their supra-normal lipreading ability^16,28,31–33,43^. Behavioural performance of CI users in the current study surprisingly disagrees with these reports and did not find better lipreading performance or confidence in responses for monosyllabic words compared to the age-matched controls. Past work has also shown that CI users with longer durations of deafness and earlier ages of deafness onset have higher lipreading performance^16,31^, but we did not find that here. Participants who used their CI for longer durations however did have higher lipreading ability, consistent with recently published findings^43^.

While these disagreements are difficult to rectify, we compared characteristics of deafness and CI use in this study to those of two closely matched studies using monosyllabic words, Stropahl et al.^16^ and Stropahl and Debener^31^ (Table 1 in both studies). Despite differences in test materials and language, lip reading performance was comparable between these three studies, with CI users performing at an average of 38% (SE = 3.8)^16^, 33% (SE = 4.0)^31^, and here, slightly lower at 31% (SE = 4.1). NH listeners scored on average 27% (SE = 4.3)^16^, 23% (SE = 3.0)^31^, and in in this report, 24% (SE = 3.1) We may not have found significant differences due to a lower number of participants (N = 28 here) versus Stropahl et al.^16^ (N = 42) and Stropahl and Debener^31^ (N = 35), and the latter two studies used one-tailed tests. In addition, our participants did not significantly differ from those reports in age or age at deafness onset, but participants in the present study had significantly longer periods of deafness (M = 31 years) compared to Stropahl et al.^16^ (~ 7 years) or Stropahl and Debener^31^ (~ 5 years; p’s < 0.001) and tended to use the CI for longer durations compared to Stropahl et al.^16^ (p = 0.052). It is possible that longer durations of CI use may have revealed the relationship we found to lipreading performance, or that lipreading performance changes complexly with longer deafness durations. We could not test for the association of task performance with short deafness durations because 75% of our sample had deafness longer than the maximum duration reported in those studies (20 years). We add that duration of deafness is difficult to quantify as onset of deafness is commonly gradual and not often instantaneous. Other sources of disagreement could be the differences in the lipreading tasks and that our stimulus did not feature a full face, but only the bottom half. Masking the top half introduces artificiality and may preclude CI users from integrating information across full-face features. These possibilities require further testing.

### Visual crossmodal activation in CI users

Past research generally suggests an upregulation of visual processing in CI users as shown by a visual bias or enhanced gain in multisensory integration or improved visual-only processing^16,28,31–33,43–47^. While intramodal plasticity within visual cortex may account for these perceptual differences^20^, crossmodal plasticity likely plays a role^20,48^. A chronic reliance on visual cues likely contributes to differences in visual crossmodal activation of auditory areas during face onset and during lip movement that we found between CI and NH groups, and furthermore may explain the relationship of visual crossmodal activation to SIN listening ability. We consider this in detail below.

CI users had stronger activation to the onset of the movie clip in left auditory cortex, but no interaction was found to suggest that the group difference was specific to this region of interest. The results differ from past studies that show higher right but not left hemisphere activation to faces in CI users^16,31^. Interestingly, CI users do not have larger crossmodal activations to pictures of houses compared to controls^16^, suggesting that crossmodal activation was specific to a stimulus that is relevant for audiovisual speech communication. Because our stimuli were not full-face but rather only the lower half of the face, crossmodal activation to the video onset in CI users may not have been as strong, or perhaps were left lateralized if the mouth region activated visual speech networks in left temporal areas^49^. Future studies might test CI users’ full-face responses versus partial-face responses to confirm this assumption.

Past research suggests that larger auditory cortical responses to faces are linked to perceptual abilities relevant to audiovisual communication. CI users with stronger crossmodal responses to faces experience more frequent fusion of McGurk stimuli^31^ and recognize faces with higher accuracy^16^. We did not assess audiovisual integration or face perception in our participants but found that CI users with higher SIN scores had stronger crossmodal activation to the onset of the video clip which featured the bottom half of a face. Considering all results, our findings are consistent with a view of cooperation between visual plasticity and audiovisual speech communication outcomes^25–27^. When interpreting our correlational findings in this framework, it is sensible that CI users are more likely to have auditory speech perception that is biased by visual speech cues if they effectively leverage visual input from facial features, and this visual bias may assist speech-in noise-perception^50^. We would then expect to see stronger face- (or partial face-) evoked crossmodal activation in auditory areas in these individuals. The effects could arise from deprivation-related crossmodal reorganization itself, plasticity related to long-term training on visual speech cues that occurred during deafness or CI rehabilitation^49^, or a combination of these forms of plasticity^3^ as has been demonstrated in amputees^51^.

Perception of lip movement is not only supported by visual regions, it is also known to involve auditory cortex in typical hearing listeners^49,52–54^. Visual language, such as sign language and speechreading, is also known activate superior temporal cortex in deaf individuals^19,55^ and CI users ^25,33^. Here we present a new finding where CI users showed a mixture of alpha rhythm synchronization and desynchronization in auditory cortex and broadly in temporal regions during lip movement. Alpha responses in NH listeners favoured desynchronization during this time. The functional meaning of alpha power changes measured using EEG or magnetoencephalography (MEG) has been debated as either reflecting activity in inhibitory circuits that operate to suppress competing information and is directed by cognitive control mechanisms^30,56^ while others have suggested that alpha oscillations may reflect stimulus enhancement in spatial processing^57^ (see also^58^). Alpha rhythms appear to modulate in a task-relevant manner consistent with participant goals and are linked to attentional processes^59^. To our knowledge, no study has examined alpha activity in auditory areas as it relates to cross-modal visual input, and the precise function of alpha oscillations in our study design is not clear. Based on the role of alpha rhythms in perceptual processing, contrasting ERS and ERD patterns in CI users and NH listeners may reflect differences in how participants processed facial features or directed their attention to speech-related lip movement in brain areas that are sensitive to visual language.

Only a few studies have compared crossmodal changes to oscillations in deafness or CI use. With orthographic visual stimuli, our group found stronger alpha ERD after stimulus onset across parietal scalp regions in CI users that may reflect stronger intramodal responses, but this activity did not correlate to SIN ability^29^. Early deaf individuals show reduced parietal scalp ERD in alpha and beta (15–25 Hz) rhythms for novel visual events (morphing circles) compared to NH controls, interpreted as a modification to automatic deviance detection for visual events in early deafness, or changes in the deployment of visual attention or inhibition^60^. These findings only suggest that CI users or deaf participants are expected to differ from control groups when measuring alpha rhythms in visual perception, but task- and stimulus-based differences make synthesis of findings difficult.

A clue to understanding the meaning of ERS/D differences is the negative correlation between these responses and SIN performance in CI users. On one hand, the correlation could be interpreted in a competition framework where visual stimulus processing related to lip movement has co-opted auditory temporal areas and prevents neurons in these regions from contributing to speech processing^14,15,22–24^. However, considering the positive association between face-evoked crossmodal activation and SIN listening in this study, and with prior work suggesting that visual plasticity in the auditory cortex does not impair auditory responsiveness^26^ and has functional benefits for auditory or audiovisual speech^25,31^, an alternative view concerns how CI users with limited SIN ability leverage visual cues in communication.

Hemodynamic responses to visual speech in typical hearing adults arise in a network that includes auditory cortical regions in temporal cortex^52–54^. One possibility is that modulations of alpha rhythms in auditory ROIs in this study reflect levels of activity in these visual speech networks, which may or may not implicate crossmodal reorganization. These levels of activity may reflect the strength or degree to which CI users rely on visual information in audiovisual speech perception. Past studies show CI users with lower speech outcomes are more likely to rely on visual input to resolve conflict in audiovisual stimuli^45^ and it is well known that visual cues can facilitate speech understanding in noisy backgrounds^50^. Recall that CI users in our study with lower SIN performance did not have evidence of stronger face-evoked auditory cortical responses, which runs counter to the competition viewpoint of crossmodal plasticity. If we assume forms of crossmodal reorganization are beneficial, then CI users with less cortical reorganization (i.e., weaker cortical activation to face onset) may more frequently rely on visual cues, like lip movement, in noisy situations. Alpha synchronization observed in lower-performing CI users’ auditory cortical regions may reflect differences in the way that they process speech-related lip movement in visual speech networks along temporal cortex because of continual assistance from visual speech cues, especially in noise. Alpha ERS could arise from a form of stimulus selection where lip movement representations are facilitated and other facial motion is suppressed (c.f.^57,61,62^) or inhibition of speech-related lip motion representations in auditory areas^30^ in a way that may favor representations of visual information in visual cortical areas^20^. A caveat to this latter alternative is that alpha ERS/D between visual and auditory areas was unrelated in CI users (p’s > 0.3) and therefore responses in visual areas were not enhanced if ERS in temporal areas is related to a form of neural suppression. ERS found in lower-performing CI users may also reflect differences in the way that these individuals focus attention or engage attention networks to facial features^59,62^. If alpha ERS reflects inhibition in sensory or cognitive networks, lower-performing CI users with this signature may not be effectively processing visual speech information in multisensory regions. While we cannot resolve these alternatives here, they invite interesting new directions for future work. At minimum, we interpret alpha ERS/ERD in auditory temporal areas in this study reflecting a form of stimulus selection or attention-related processing when participants observe speech-related lip movement, and individual differences in these processes relate to different levels of speech-in-noise listening performance in CI users.

## Conclusion

Past studies have generally compared visual crossmodal activation of auditory cortex to speech outcomes in CI users by assessing individual differences to one stage of visual stimulus processing or one type of visual event. These studies have found negative correlations providing evidence in support of a competition view where crossmodal reorganization is associated with poorer speech outcomes, or positive correlations supporting a cooperation view where visual plasticity works synergistically to restore speech ability after cochlear implantation. This discrepancy is important to resolve, because there have been recommendations to limit exposure to visual language if it negatively affects speech outcomes (for instance, see^63^), but this in fact may be counterproductive for CI rehabilitation^25^. Here we found both positive and negative correlations between speech-in-noise perception and visual crossmodal activations in the same group of CI users. If one were to examine one crossmodal response (e.g., lip movement) over the other (face onset), different conclusions may have been drawn. Considering the data and supporting evidence across multiple studies, we argue that crossmodal reorganization relates to improved speech outcomes, but stimulus-dependent types of crossmodal activations could reflect differences in perception or attention to visual speech in higher- and lower- performing CI users.

## Data Availability

The datasets generated during and/or analyzed during the current study will be available from the Open Science Framework website upon publication (https://osf.io/xnjzg/).
